# Procedure of the overexpression, purification and crystallization of BLEG-1, a bifunctional and evolutionary divergent B3 metallo-β-lactamase, for structure-function studies

**DOI:** 10.1016/j.mex.2022.101740

**Published:** 2022-05-27

**Authors:** Shaw Xian Au, Noor Dina Muhd Noor, Hiroyoshi Matsumura, Raja Noor Zaliha Raja Abdul Rahman, Yahaya M. Normi

**Affiliations:** a Enzyme and Microbial Technology (EMTech) Research Center, Faculty of Biotechnology and Biomolecular Sciences, Universiti Putra Malaysia, Serdang, Selangor 43400, Malaysia; b Department of Cell and Molecular Biology, Faculty of Biotechnology and Biomolecular Sciences, Universiti Putra Malaysia, Serdang, Selangor 43400, Malaysia; c Department of Biochemistry, Faculty of Biotechnology and Biomolecular Sciences, Universiti Putra Malaysia, Serdang, Selangor 43400, Malaysia; d College of Life Sciences, Ritsumeikan University, Noji-Higashi, Kusatsu 525-8577, Japan; e Department of Microbiology, Faculty of Biotechnology and Biomolecular Sciences, Universiti Putra Malaysia, Serdang, Selangor 43400, Malaysia

**Keywords:** BLEG-1, B3 metallo-β-lactamase, Protein production, Protein purification, Protein crystallography

## Abstract

Metallo-β-lactamases (MBLs) are class B β-lactamases from the metallo-hydrolase-like MBL-fold superfamily which act on a broad range of β-lactam antibiotics, thus conferring antibiotics resistance to bacterial pathogens. The attempt to structurally characterize BLEG-1, an evolutionary divergent B3 metallo-β-lactamase (MBL) with dual activity from *Bacillus lehensis* G1, led to the optimization of its purification, post-purification and crystallization processes for X-ray diffraction purpose. The workflow, conditions used and dataset obtained from each stage of the processes are presented herein. The optimization workflow has enabled the obtainment of purified, active BLEG-1 in high yield for its activity assays, crystallization and structure determination via X-ray diffraction. This is the first step to gain a better insight into its dual activity and evolutionary divergence from a structural perspective. The complete research article, including BLEG-1 dual activity analysis, is published in the International Journal of Molecular Sciences (Au et al., 2021).

• The method was optimized to increase the stability of BLEG-1 in purification, post-purification and crystallization processes.

• Protein crystallization using the optimized conditions presented herein is able to produce and regenerate BLEG-1 protein crystals of medium-size, which is an advantage in X-ray diffraction.

• The method can be used for relevant homologs and variants of BLEG-1 for structure-function and mechanistic studies of such proteins.

## Graphical abstract

**Figure:**
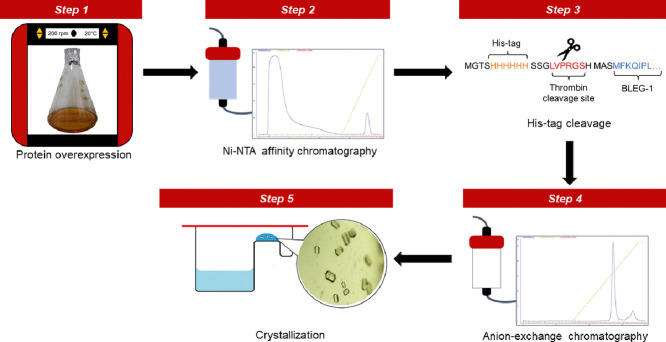

Specifications table

**Table:** 

**Subject Area**	Biochemistry, Genetics and Molecular Biology
**More specific subject area**	Structural biology
**Method name**	Optimized and regenerable two-steps purification and crystallization of evolutionary divergent B3 metallo-β-lactamase
**Name and reference of original method**	S.X. Au, N.S. Dzulkifly, N.D.M. Noor, H. Matsumura, R.N.Z.R.A. Rahman, Y.M. Normi, Dual Activity BLEG-1 from *Bacillus lehensis* G1 Revealed Structural Resemblance to B3 Metallo-β-Lactamase and Glyoxalase II: An Insight into Its Enzyme Promiscuity and Evolutionary Divergence, Int. J. Mol. Sci. 22 (2021) 9377. https://doi.org/10.3390/ijms22179377
**Resource availability**	Not applicable.

## Background

The production of β-lactamases is one of the common mechanisms that bacteria employ to inactivate the action of β-lactam antibiotics by cleavage amide bond of the β-lactam ring of the antibiotics before they reach the target site in bacteria. There are two types of β-lactamases, which are serine-β-lactamases (SBLs) and metallo-β-lactamases (MBLs). MBLs or class B β-lactamases belong to the metallo-hydrolase-like MBL-fold superfamily, which consists a large and ancient group of proteins with diverse catalytic functions. MBLs are characterized by the αβ/βα protein fold and the His-Xaa-His-Xaa-Asp-His motif that coordinates zinc ion(s) for their catalytic reaction [2]. There are three subclasses of MBLs: B1, B2, and B3. B3 subclass is phylogenetically distinct from the B1 and B2 subclasses despite their substrate specificity, and was considered as a separate MBL group [3]. It was suggested that convergent evolutionary events within the MBL family involved the alterations of metal binding motif and active site configuration of the proteins, which led to the emergence of MBLs fostering the same functionality through different mechanisms [4], [5], [6]. Due to the low structural similarity and unique active sites of B1, B2 and B3 MBLs, urgent search of useful clinical inhibitors which can act specifically on the proteins is in demand. Since the past decade, a number of promising inhibitors targeting on MBLs were discovered, such as aspergillomarasmine [7], thiol-based compounds [8], and phosphonate-containing compounds [9]; with most inhibiting the proteins by chelating and/or binding to their zinc ligands. However, most of the inhibitors are still in the pre-clinical stage and require further improvement [10]. This can be accelerated by *in silico* structure-based drug design approach coupled with their *in vitro* analyses. In addition to this, discoveries of B3 MBLs with bifunctional properties have set forth scientific discourse and investigations on the evolution of β-lactamases. B3 MBLs have been reported to be functionally and phylogenetically diverse [5]. A case in point is PNGM-1 isolated from deep sea sediment metagenome that exhibited both MBL and tRNase Z activities. The crystal structure of PNGM-1 revealed that the enzyme adopted structural features which are crucial for the binding of tRNA and β-lactam antibiotics, suggesting it could be an intermediate in the evolutionary path of B3 MBL from tRNase Z [11]. To date, more putative MBLs with enzymatic promiscuity have been identified that have yet to be structurally characterized to probe into their dual functionality [6]. Hence, in-depth understanding of the structure-function and mechanistic properties of MBLs is necessary not only to facilitate the search and design of promising inhibitors but to understand their evolution in acquiring antibiotic-deactivating properties as well.

There are several methods that can be used to determine the three-dimensional (3D) atomic structure of protein, which are the X-ray crystallography, nuclear magnetic resonance (NMR) spectroscopy, and cryo-electron microscopy (cryo-EM). According to the statistics in the Protein Data Bank (PDB), X-ray crystallography is still the predominant method in protein structure determination to date (https://www.rcsb.org/stats/all-released-structures, accessed on 7 Dec 2021). This is a well-established method which is able to yield high resolution data and is not limited by protein size [12]. The obtainment of protein crystals of good quality (i.e. single crystal with medium-to-large size and of appropriate shape) for diffraction is often desired, as this would increase the probability of obtaining diffraction data and eventually a protein structure of higher quality [13]. For this purpose, it is necessary to produce protein sample at high concentration, homogeneity, solubility and stability. Protein stability is known as the basic prerequisite in X-ray crystallography. At the overexpression and purification stage, protein stability ensures protein folds into its native conformation, as opposed to the formation of protein aggregates, which would eventually affect protein yield and function. In subsequent crystallization step, protein stability favours the self-arrangement of protein molecules into a regular order, hence facilitating the formation of good crystals. There are several factors that affect protein stability, which should be considered into the experimental design. These include the chemical (i.e. amino acids) and structural (i.e. α-helices, β-sheets, loops) compositions of protein, dynamic variability of protein domains, environment and conditions (i.e. buffer composition, pH, temperature etc) [14]. Protein stability can be improved by a proper design of expression construct, and optimization of environment and conditions. For instance, the addition of metal ligands into cultivation media promote proper protein folding during expression [15]; while stabilizing additives such as glycerol and polyethylene glycol (PEG) can enhance protein stability during crystal formation [14].

The methods presented herein focused on the large-scale production of BLEG-1 B3 MBL at high homogeneity and stability for the crystallization of BLEG-1. The method is optimized and reproducible, with most details on the optimization of buffer components and conditions shown. This method can serve as a foundation for further manipulations, to prepare BLEG-1 and other homologs for structure-function studies.

## Method details

*Step 1: BLEG-1 overexpression*

*Materials and apparatus*

• Luria-Bertani (LB) broth (25 g Difco^TM^ LB broth, Miller in 1 L distilled water)
• 50 mg/mL kanamycin
• 1 M isopropyl-β-D-thiogalactopyranoside (IPTG)
• 1 M ZnSO_4_
• 40 mL Bijou bottle
• 5 L Erlenmeyer flask
• 50 mL centrifuge tube
• Ecotron incubation shaker (Infors AG, Switzerland)
• Varian Cary® 50 UV-Vis spectrophotometer (Agilent Technologies, USA)
• Refrigerated centrifuge 5804R (Eppendorf, Germany)

*Procedure*

1. Based on the method by Selvaraju et al. [16] with slight modifications, *Escherichia coli* BL21(DE3) cells harbouring pET-28b(+)::*Bleg-1* recombinant plasmid (from glycerol stock) were cultivated in 120 mL sterile LB broth containing 50 μg/mL of kanamycin at 37˚C for 18 h, in an incubation shaker with agitation speed of 200 rpm.
2. 120 mL of the starter culture was used to inoculate into 3 L sterile LB broth containing 50 μg/mL of kanamycin and 0.1 mM ZnSO_4_. The culture was cultivated at 37˚C with agitation at 200 rpm.
3. When the optical density at 600 nm (OD_600_) of the bacterial culture reached 0.6, 0.1 mM of IPTG was added, followed by further cultivation at 20˚C for 20 h, with agitation at 200 rpm. The cells were collected by centrifugation at 9,000 x g, at 4˚C for 20 min.

*Step 2: First-step BLEG-1 purification via Ni-NTA affinity chromatography*

*Materials and apparatus*

• Buffer 1 (20 mM Na_2_HPO_4_–NaH_2_PO_4_ buffer, 500 mM NaCl, 50 mM imidazole, 2 mM MgSO_4_•7H_2_O; pH 7.4)
• Buffer 2 (20 mM Na_2_HPO_4_–NaH_2_PO_4_ buffer, 500 mM NaCl, 500 mM imidazole; pH 7.4)
• Buffer 3 (20 mM Na_2_HPO_4_–NaH_2_PO_4_ buffer, 20 mM NaCl; pH 7.4)
• Bradford reagent (Sigma-aldrich, USA)
• Bovine serum albumin (BSA)
• Syringe
• Syringe filter with 0.45 µm cellulose acetate membrane (Sartorius, Germany)
• 5 mL pre-packed HisTrap^TM^ FF column (GE Healthcare, USA)
• SnakeSkin Dialysis Tubing, 10 kDa MWCO (Thermo Fisher Scientific, USA)
• 15 mL centrifuge tube
• 5 L Erlenmeyer flask
• Plastic cuvette
• Refrigerated centrifuge 5804R (Eppendorf, Germany)
• Q55 Sonicator with 1/4’’ (6mm) probe (Qsonica, USA)
• ÄKTAPrime plus system (GE Healthcare, USA)
• Mini-PROTEAN tetra cell vertical gel electrophoresis system (Bio-Rad Laboratories, USA)
• Varian Cary® 50 UV-Vis spectrophotometer (Agilent Technologies, USA)

*Procedure*

1. Cell pellet collected from 3 L bacterial culture in step 1 was resuspended in buffer 1, and lyzed using a sonicator with 1/4’’ (6mm) probe, at 40% amplitude, 15 s ON and 15 s OFF pulses, for 12 min, on ice.
2. The cell lysate was then centrifuged at 9,000 x g, 4˚C for 20 min, in which the supernatant was subsequently passed through a 0.45 µm cellulose acetate membrane by syringe filtration.
3. The filtered supernatant was loaded into a 5 mL pre-packed HisTrap^TM^ FF column at flowrate of 1.0 mL/min, which was connected to ÄKTAPrime plus system and pre-equilibrated with buffer 1.
4. His-tagged protein bound on the resin was washed with 20 column volume (CV) of buffer 1 and eluted in a linear gradient of imidazole (50–500 mM imidazole) using 20 CV of buffer 2.
5. The elution fractions were collected, analyzed by SDS-PAGE (12%), pooled in a dialysis tubing and dialyzed against 4 L of buffer 3 (i.e. protein-to-buffer ratio of 1:160), at 4°C for 18 h.
6. Concentrations of protein were determined by Bradford assay [17], using Bradford Reagent (Sigma-aldrich, USA) with bovine serum albumin as standard and measured at 595 nm using Varian Cary® 50 UV-Vis spectrophotometer (Agilent Technologies, USA).

*Step 3: Optimization of His-tag cleavage*

*Materials and apparatus*

• Buffer A (20 mM Na_2_HPO_4_–NaH_2_PO_4_ buffer, 0.1 mM ZnSO_4_, 5% (v/v) glycerol; pH 7.4)
• Buffer B (20 mM Na_2_HPO_4_–NaH_2_PO_4_ buffer, 0.1 mM ZnSO_4_; pH 7.4)
• SnakeSkin Dialysis Tubing, 10 kDa MWCO (Thermo Fisher Scientific, USA)
• 1 U/µL thrombin
• 5 L Erlenmeyer flask
• 1.5 mL microcentrifuge tube
• Waterbath WNB 14 (Memmert, Germany)
• Mini-PROTEAN tetra cell vertical gel electrophoresis system (Bio-Rad Laboratories, USA)

*Procedure*

1. Purified His-tagged fusion BLEG-1 obtained via Ni-NTA affinity chromatography was dialyzed using similar method mentioned in step 2 with buffer A and buffer B, respectively.
2. One mg/mL of the dialyzed protein sample was then added with 1 U/µL of thrombin enzyme, and incubated in waterbath at 4°C and 25°C, for 24 h.
3. The mixtures were aliquoted at time intervals of 0, 2, 4, 8, 16 and 24 h, and subjected to SDS-PAGE (12%) analysis.

*Step 4: His-tagged cleavage and second-step BLEG-1 purification via anion-exchange chromatography*

*Materials and apparatus*

• 1 U/µL thrombin
• Buffer 3 (20 mM Na_2_HPO_4_–NaH_2_PO_4_ buffer, 20 mM NaCl; pH 7.4)
• Buffer 4 (20 mM Na_2_HPO_4_–NaH_2_PO_4_ buffer, 500 mM NaCl; pH 7.4)
• Buffer 5 (20 mM Na_2_HPO_4_–NaH_2_PO_4_ buffer, 100 mM NaCl, 0.1 mM ZnSO_4_, 5% (v/v) glycerol; pH 7.4)
• 5 mL Q-Sepharose^TM^ FF packed in XK16/20 column (GE Healthcare, USA)
• Bradford reagent (Sigma-aldrich, USA)
• SnakeSkin Dialysis Tubing, 10 kDa MWCO (Thermo Fisher Scientific, USA)
• 15 mL centrifuge tube
• 5 L Erlenmeyer flask
• Waterbath WNB 14 (Memmert, Germany)
• ÄKTAPrime plus system (GE Healthcare, USA)
• Mini-PROTEAN tetra cell vertical gel electrophoresis system (Bio-Rad Laboratories, USA)
• Varian Cary® 50 UV-Vis spectrophotometer (Agilent Technologies, USA)

*Procedure*

1. 1 U/µL of thrombin enzyme was added to per mg/mL of dialyzed His-tagged fusion BLEG-1 obtained in step 2, and incubated at 25°C for 16 h.
2. BLEG-1 with and without the addition of thrombin were analyzed by SDS-PAGE (12%).
3. Thrombin-treated BLEG-1 was loaded into a 5 mL Q-Sepharose^TM^ FF column at flowrate of 1.0 mL/min, which was connected to ÄKTAPrime plus system and pre-equilibrated with buffer 3.
4. The bound protein was washed with 12 CV of buffer 3 and eluted in a linear gradient of NaCl (20–500 mM NaCl) using 20 CV of buffer 4.
5. The eluates were collected and analyzed via SDS-PAGE (12%), pooled and dialyzed against 4 L of buffer 5 at 4°C for 18 h using the same dialysis tubing and protein-to-buffer ratio as mentioned in Step 2.
6. Concentrations of protein were determined using the same method in Step 2.

*Step 5: BLEG-1 crystallization and optimization*

*Materials and apparatus*

• Vivaspin 6, 10 kDa MWCO Polyethersulfone centrifugal concentrator (Sartorius, Germany)
• Crystal Screen 1^TM^ and Crystal Screen 2^TM^ (Hampton Research, USA)
• PEG/Ion Screen 1^TM^ and PEG/Ion Screen 2^TM^ (Hampton Research, USA)
• Index Screen^TM^ (Hampton Research, USA)
• JCSG plus^TM^ (Molecular Dimensions, UK)
• NaI
• 50% (w/v) Polyethylene glycol (PEG) 3,350 Monodisperse
• INTELLI-PLATE® 96 Well plates (Art Robbins Instruments, USA)
• Crystal clear sealing tape (Hampton Research, USA)
• Refrigerated centrifuge 5804R (Eppendorf, Germany)
• Oryx8 protein crystallization robot (Douglas Instruments, UK).
• EchoTherm^TM^ chilling incubator (Torrey Pines Scientific, USA)

*Procedure*

1. For pre-screening of BLEG-1 concentration for crystallization, purified BLEG-1 was concentrated to 3 mg/mL and 17 mg/mL via centrifugation with speed of 8,000 x g at 4°C, using Vivaspin 6 centrifugal concentrator with MWCO of 10 kDa.
2. Pre-screening of the crystallization formulations and conditions for BLEG-1 were conducted using the commercial crystallization screening kits as mentioned in the “materials and apparatus” section.
3. Crystallization was carried out on INTELLI-PLATE® 96 Well crystallization plates that were pre-filled with 240 µL of crystallization formulations in the reagent reservoirs, using 3 and 17 mg/mL of purified BLEG-1, through sitting-drop vapour diffusion method by Oryx8 protein crystallization robot.
4. By mixing 1 µL of protein with 1 µL and 0.5 µL of formulation respectively, droplets of 1:1 and 2:1 (protein-to-formulation) ratio were made in the drop wells of crystallization plates.
5. The crystallization plates were sealed and incubated at 4°C.
6. Subsequent optimization of BLEG-1 crystallization conditions was performed by varying the concentrations of NaI (i.e. 0.1, 0.2, 0.3, 0.4, and 0.5 M) and PEG3350 (i.e. 10%, 20%, and 30% (w/v)), using 17 mg/mL of purified BLEG-1, at 1:1 protein-to-formulation ratio, and incubated at 4°C and 20°C.

## Methods validation

**Fig. 1:**
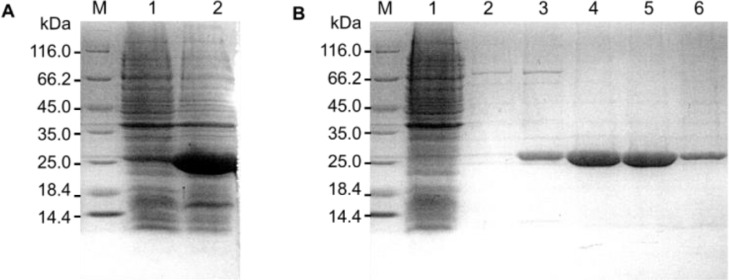
SDS-PAGE (12%) gel image of BLEG-1 His-tagged fusion protein (26 kDa) (A) expressed in insoluble fraction (Lane 1), soluble fraction (Lane 2); and (B) purified via affinity chromatography (Lane 1: flow-through, Lane 2-6: purified fractions.). Lane M: unstained protein marker (Thermo Fisher Scientific, USA), size range: 14.4–116.0 kDa.

The target enzyme, BLEG-1, carrying an N-terminal His-tag was overexpressed in soluble form in *E. coli* BL21(DE3) after cultivation in LB broth with 0.1 mM IPTG induction at 20˚C for 20 h. BLEG-1 recombinant protein was purified to near homogeneity using Ni-NTA affinity chromatography (Fig. 1).

**Fig. 2:**
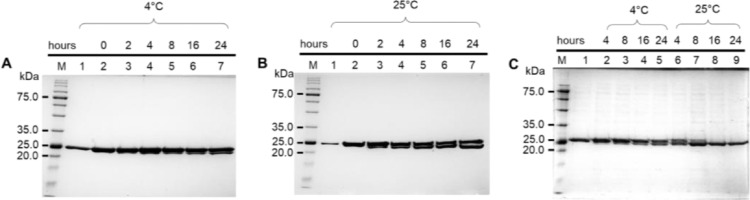
SDS-PAGE (12%) gel image of His-tagged fusion BLEG-1 treated with thrombin in the presence of glycerol at (A) 4 °C; (B) 25 °C; and (C) in the absence of glycerol at 4 °C and 25 °C, at various incubation period. Lane M: BLUelf prestained protein ladder (GeneDireX, Inc, USA), size range: 5.0–245.0 kDa; Lane 1: purified BLEG-1 without the addition of thrombin (control); Lane 2 onwards: BLEG-1 treated with thrombin at different incubation period. The sizes of BLEG-1 with and without His-tag were 26 kDa and 23 kDa, respectively.

Subsequent His-tag cleavage which was initially performed in a buffer containing glycerol for protein stabilization showed incomplete cleavage of His-tag from BLEG-1 at both 4°C and 25°C (Fig. 2A, B), using 1 U/µL of thrombin enzyme per mg/mL of His-tagged fusion BLEG-1. It was hypothesized that the presence of 5% (v/v) glycerol could have lowered the catalytic activity of thrombin and led to incomplete cleavage of His-tag in BLEG-1 [18]. Without the presence of glycerol in the buffer, complete cleavage of His-tag from BLEG-1 was achieved after 16 h of incubation at 25°C, using the same amount of thrombin as mentioned above (Fig. 2C).

**Fig. 3:**
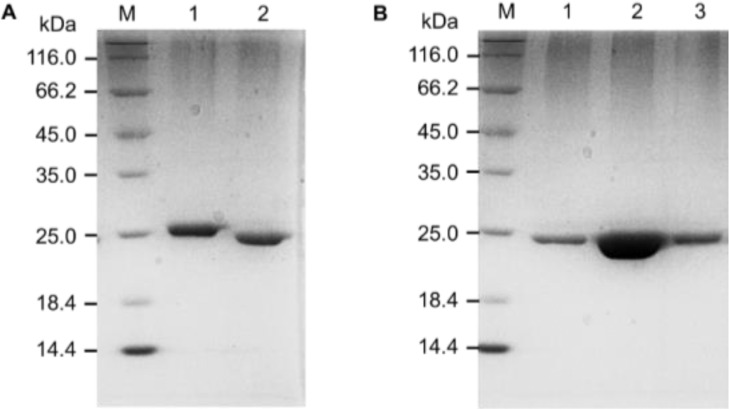
SDS-PAGE (12%) gel image of cleaved BLEG-1 purified via (A) affinity chromatography (Lane 1: BLEG-1 without His-tag cleavage (control); Lane 2: BLEG-1 after His-tag cleavage); and (B) anion-exchange chromatography (Lane 1–3: purified fractions of His-tag-cleaved BLEG-1). Lane M: unstained protein marker (Thermo Fisher Scientific, USA), size range: 14.4–116.0 kDa. The sizes of BLEG-1 with and without His-tag were 26 kDa and 23 kDa, respectively.

To maintain the stability of BLEG-1 for dialysis and second-step purification, 20 mM NaCl was added into the buffer (Table 1), and this had no effect on His-tag cleavage of BLEG-1 under the optimized conditions (Fig. 3A, Lane 2). BLEG-1 without His-tag was purified to homogeneity using anion-exchange chromatography (Fig. 3B, Lane 1–3), which is an important requirement for protein crystallization.

**Table 1:** Compilation of optimization parameters to date for the production and purification of BLEG-1 B3 MBL.

Details	Tan et al. [**19**]	Selvaraju et al. [**16**]	Au et al. [**1**]	Remarks
**Expression host**	*E. coli* Rosetta-gami (DE3)	*E. coli* BL21(DE3)	*E. coli* BL21(DE3)	
**Expression plasmid**	pET-32b	pET-28b	pET-28b	
**Cultivation medium and volume**	Luria-Bertani broth 200 mL	Luria-Bertani broth 3 L	Luria-Bertani broth 3 L	
**Antibiotics in cultivation medium**	50 μg/mL ampicillin and 34 μg/mL chloramphenicol 200 mL medium	50 μg/mL kanamycin 3 L medium	50 μg/mL kanamycin 3 L medium	
**100 µM ZnSO_4_ in cultivation medium**	Absent	Present	Present	100 µM ZnSO_4_ was added to the cultivation medium as a means to allow the target protein to be incorporated with Zn^2+^.
**Method of purification and buffers used**	*Affinity chromatography* Buffer 1: 20 mM Na_2_HPO_4_•7H_2_0, 1 M NaCI (pH 7) Buffer 2: 20 mM Na_2_HPO_4_•7H_2_O, 1 M NaCI, 1 M imidazole (pH 7)	*Affinity chromatography* Buffer 1: 20 mM Na_2_HPO_4_–NaH_2_PO_4_, 500 mM NaCl, 50 mM imidazole, 2 mM MgSO_4_ (pH 7.4) Buffer 2: 20 mM Na_2_HPO_4_–NaH_2_PO_4_, 500 mM NaCl and 500 mM imidazole (pH 7.4)	*Affinity chromatography* Buffer 1: 20 mM Na_2_HPO_4_–NaH_2_PO_4_, 500 mM NaCl, 50 mM imidazole, 2 mM MgSO_4_•7H_2_O (pH 7.4) Buffer 2: 20 mM Na_2_HPO_4_–NaH_2_PO_4_, 500 mM NaCl, 500 mM imidazole (pH 7.4) *Anion exchange chromatography* Buffer 3: 20 mM Na_2_HPO_4_–NaH_2_PO_4_, 20 mM NaCl, (pH 7.4) Buffer 4: 20 mM Na_2_HPO_4_–NaH_2_PO_4_, 500 mM NaCl (pH 7.4)	Buffer composition used in Selvaraju et al. [16] and Au et al. [1] resulted in less protein aggregation post purification, with the addition of 500 mM NaCl.
**His-tag cleavage**	No	No	Yes	
**Method for buffer exchange, protein concentration and buffer used**	*Ultrafiltration* Buffer 3: 10 mM Na_2_HPO_4_•7H_2_O (pH 7). 5% (v/v) glycerol was later added to the protein sample.	*Dialysis* Buffer 3: 20 mM Na_2_HPO_4_– NaH_2_PO_4_, 100 µM ZnSO_4_, and 5% (v/v) glycerol (pH7.4)	*Dialysis 1* Buffer 3: 20 mM Na_2_HPO_4_–NaH_2_PO_4_, 20 mM NaCl, (pH 7.4) *Dialysis 2* Buffer 5: 20 mM Na_2_HPO_4_–NaH_2_PO_4_, 100 mM NaCl, 100 µM ZnSO4, 5% (v/v) glycerol (pH 7.4) *Ultrafiltration* Protein sample obtained from Dialysis 2 was used.	Dialysis allows buffer exchange to take place gradually over time, in a diluted environment. This minimizes protein aggregation. For additional safe measures, ZnSO_4_ was also incorporated in the dialysis buffer in the protocol by Selvaraju et al. [16] and Au et al. [1]. The addition of 100 mM NaCl in Buffer 5 (dialysis 2) by Au et al. [1] ensures better buffering of protein for longer duration during crystallization.
**Protein concentration obtained**	3.2 mg	27 mg	32 mg

**Fig. 4:**
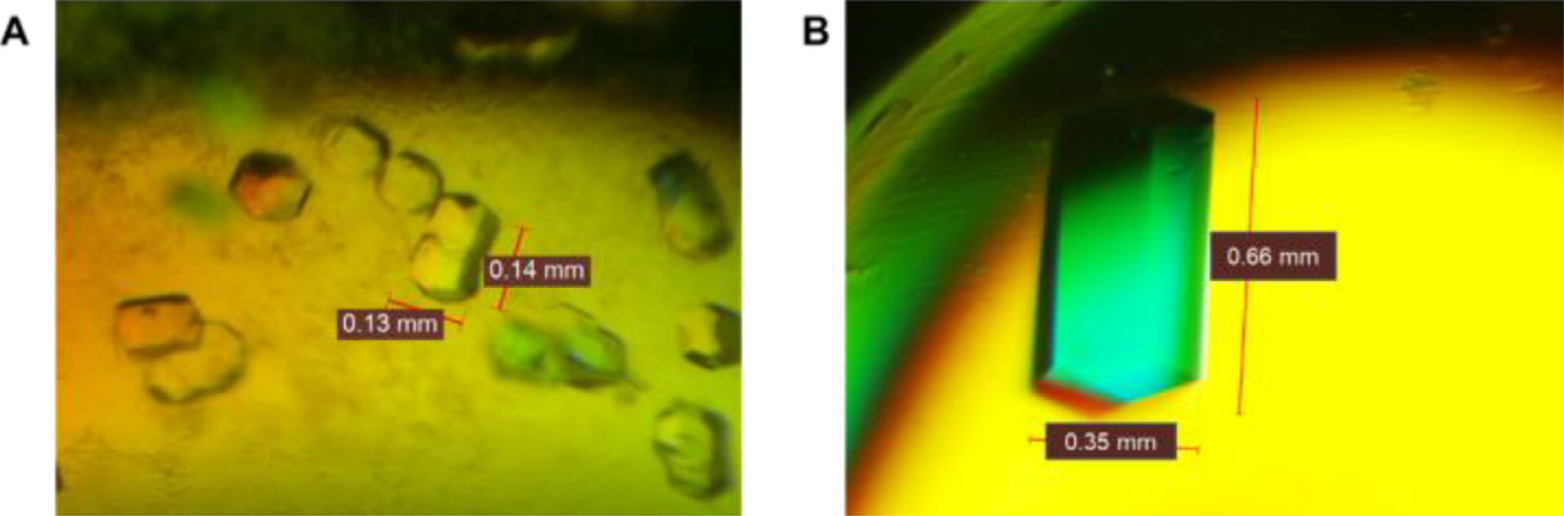
Protein crystals formed in (A) formulation Tube 10 PEG/ Ion Screen 1^TM^ (Hampton Research, USA) containing 0.2 M NaI and 20% (w/v) PEG3350 at 4 °C, and (B) in optimized formulation containing 0.5 M NaI and 30% (w/v) PEG3350 at 20 °C, at 1:1 protein-to-formulation ratio, after 14 days of incubation.

Pre-screening of crystallization conditions for BLEG-1 showed that the target enzyme crystallized at 17 mg/mL in the formulation containing 0.2 M NaI and 20% (w/v) polyethylene glycol (PEG) 3350 (i.e. Tube 10 of PEG/Ion Screen 1^TM^ (Hampton Research, USA)), in 1:1 protein-to-formulation ratio, after 14 days of incubation at 4°C (Fig. 4A, Table 2). To further improve the size and morphology of protein crystals, further optimization of the crystallization conditions was performed using the same protein concentration i.e. 17 mg/mL and protein-to-formulation ratio of 1:1, at 4°C and 20°C, respectively (Tables 2 and 3). The crystallization formulation containing 0.5 M NaI and 30% (w/v) PEG3350 at 1:1 protein-to-formulation ratio yielded distinct, single, tetragonal-shaped protein crystals of approximate size of 0.6 mm, after 14 days of incubation at 20°C (Fig. 4B, Table 3). Protein crystals with similar morphology and size (0.3–0.6 mm) were reproducible using the optimized crystallization conditions, which eventually enabled X-ray diffraction and structure determination of BLEG-1 at 1.44 Å (deposited in PDB, ID: 7EV5) [1].

**Table 2:** Observation of BLEG-1 protein crystal growth at 4 °C during optimization of crystallization conditions.

	0.1 M NaI	0.2 M NaI	0.3 M NaI	0.4 M NaI	0.5 M NaI
10% (w/v) PEG3350				-	-
20% (w/v) PEG3350					
30% (w/v) PEG3350					

“-” denotes no protein crystals formed.

**Table 3:** Observation of BLEG-1 protein crystal growth at 20 °C during optimization of crystallization conditions.

	0.1 M NaI	0.2 M NaI	0.3 M NaI	0.4 M NaI	0.5 M NaI
10% (w/v) PEG3350	-	-	-	-	-
20% (w/v) PEG3350	-	-	-	-	-
30% (w/v) PEG3350	-	-			

“-” denotes no protein crystals formed.

## Declaration of Competing Interest

The authors declare that they have no known competing financial interests or personal relationships that could have appeared to influence the work reported in this paper.
